# Unstable occult scaphoid fracture diagnosed by dynamic point-of-care ultrasound: a case report and review

**DOI:** 10.3389/fmed.2025.1711119

**Published:** 2025-11-05

**Authors:** Yong-Hyun Yoon, Jihyo Hwang, King Hei Stanley Lam, Jeimylo Cristobal De Castro, Teinny Suryadi, Anwar Suhaimi, Chun Wei Kang, Howon Lee, Hyeongjik Kim, Seungbeom Kim, Daniel Chiung-Jui Su

**Affiliations:** ^1^Kangnam Sacred Heart Hospital, Seoul, Republic of Korea; ^2^Incheon Terminal Orthopedic Surgery Clinic, Incheon, Republic of Korea; ^3^International Academy of Regenerative Medicine, Incheon, Republic of Korea; ^4^The Faculty of Medicine, The University of Hong Kong, Hong Kong, Hong Kong SAR, China; ^5^The Faculty of Medicine, The Chinese University of Hong Kong, New Territory, Hong Kong, Hong Kong SAR, China; ^6^The Board of Clinical Research, The International Association of Musculoskeletal Medicine, Kowloon, Hong Kong SAR, China; ^7^SMARTMD Center for Non-surgical Pain Interventions, Manila, Philippines; ^8^Adventist University of the Philippines, Silang, Philippines; ^9^Department of Physical Medicine and Rehabilitation, Hermina Podomoro Hospital, North Jakarta, Indonesia; ^10^Department of Physical Medicine and Rehabilitation, Medistra Hospital, South Jakarta, Indonesia; ^11^Physical Medicine and Rehabilitation, Synergy Clinic, West Jakarta, Indonesia; ^12^Department of Rehabilitation Medicine, Universiti Malaya, Kuala Lumpur, Malaysia; ^13^Taipei Medical University, Taipei City, Taiwan; ^14^Kangdong Sacred Heart Hospital, Gangdong-gu, Republic of Korea; ^15^Ahyeon Orthopedic Clinic, Seoul, Republic of Korea; ^16^Miso Pain Clinic, Suwon, Republic of Korea; ^17^Chi Mei Medical Center, Yongkang District, Taiwan; ^18^Tempo Regeneration Center for Musicians, Tainan, Taiwan

**Keywords:** scaphoid fracture, occult fracture, dynamic ultrasonography, wrist injury, point-of-care ultrasound, fracture stability

## Abstract

**Background:**

The scaphoid is the most frequently fractured carpal bone, yet its diagnosis remains a significant clinical challenge. A substantial percentage of non-displaced fractures are missed on initial radiographs, leading to delays in treatment and an increased risk of serious long-term complications such as non-union and avascular necrosis. While advanced imaging like CT and MRI are highly accurate, they are associated with higher costs, radiation exposure (CT), and limited immediate availability. High-resolution musculoskeletal ultrasound has emerged as a rapid, non-invasive, and cost-effective alternative. Its unique ability to perform dynamic, real-time assessment of fracture stability offers a significant advantage over static imaging modalities.

**Case presentation:**

A 29-year-old woman presented to our outpatient clinic with acute left wrist pain following a traction-fall injury. An initial four-view radiographic series of the wrist revealed no definitive evidence of a fracture. Despite the negative imaging, clinical suspicion remained high due to persistent, exquisite point tenderness over the anatomical snuffbox. A point-of-care musculoskeletal ultrasound examination was performed, which revealed a clear hypoechoic cortical breach at the scaphoid waist. To assess mechanical stability, a dynamic stress maneuver—defined as a gentle, controlled “heel-toe” probe rocking that applies focal pressure across the fracture—was performed under real-time sonographic visualization. Gentle probe pressure combined with passive ulnar deviation of the wrist demonstrated visible gapping and micromotion at the fracture site, confirming it as mechanically unstable. Based on this definitive finding, the diagnosis was revised to an unstable occult scaphoid waist fracture, and the management plan was immediately upgraded to a rigid thumb spica splint. Long-term follow-up over 2 years showed radiographic and sonographic evidence of a stable fibrous union.

**Conclusion:**

This case report highlights the pivotal role of dynamic musculoskeletal ultrasound as an adjunct in the diagnostic algorithm for acute wrist trauma. It demonstrates its ability not only to identify a radiographically occult scaphoid fracture but, more critically, to provide immediate functional information about mechanical stability. This information is paramount for guiding appropriate and timely management to mitigate the risk of long-term complications. We advocate for the broader integration of dynamic ultrasound into the initial assessment of suspected scaphoid fractures.

## 1 Introduction

The scaphoid bone is the most commonly fractured carpal bone, representing 60–70% of all carpal fractures and 2–7% of all skeletal fractures (1, 2). These injuries predominantly affect young, active individuals (ages 15–29) following a fall onto an outstretched hand (FOOSH), with significant socioeconomic impact including prolonged immobilization, lost work/sport time, and potential long-term disability (3, 4). The scaphoid serves as a critical mechanical link between proximal and distal carpal rows, with its complex, three-dimensional anatomy and articulations with four carpal bones (lunate, capitate, trapezium, trapezoid) enabling essential wrist motions like the dart-thrower’s motion crucial for daily and athletic activities (5, 6). During FOOSH injuries, extreme extension and radial deviation concentrate immense forces across the scaphoid’s slender waist, the site of 70–80% of scaphoid fractures (7).

Despite its prevalence, acute diagnosis of scaphoid fractures remains notoriously difficult. Between 5 and 20% of non-displaced scaphoid fractures are missed on initial four-view radiographs (8), due to the scaphoid’s complex anatomy, oblique orientation, and often subtle, non-displaced nature of initial injury. Clinical findings (anatomical snuffbox tenderness, scaphoid tubercle tenderness, thumb compression pain) are sensitive but lack specificity, leading to over-immobilization of unconfirmed suspected fractures (9). A missed or delayed diagnosis is particularly problematic due to the scaphoid’s unique, retrograde vascularity originating from the dorsal carpal branch of the radial artery. Fractures, especially of the waist or proximal pole, can disrupt blood flow, placing the proximal fragment at high risk of avascular necrosis (AVN) and non-union (10, 11). Scaphoid non-union leads to progressive carpal instability (DISI pattern) and, over years, to scaphoid non-union advanced collapse (SNAC wrist), a debilitating pattern of post-traumatic osteoarthritis often requiring complex surgical salvage (12–14).

The conventional diagnostic algorithm involves cast immobilization followed by repeat radiographs in 10–14 days, but this delays diagnosis and necessitates unnecessary immobilization (15). Advanced imaging (CT, MRI) is highly accurate but has significant drawbacks: radiation exposure (CT), high cost, and limited availability in acute settings (16–18). High-resolution musculoskeletal ultrasonography (MSK-US) has emerged as a cost-effective, radiation-free, point-of-care alternative with high sensitivity and specificity for detecting scaphoid cortical breaches (19–23). However, while static ultrasound identifies fracture presence, dynamic ultrasonography uniquely provides real-time, functional assessment of fracture stability under controlled stress—the single most critical factor determining management, as unstable fractures have markedly higher non-union risk when treated non-operatively (24, 25). Despite its potential, reports demonstrating how dynamic ultrasound findings decisively alter treatment pathways remain limited. This case illustrates diagnosis of a radiographically occult scaphoid fracture classified as mechanically unstable using dynamic point-of-care ultrasound, demonstrating how definitive functional information led to immediate management escalation.

## 2 Case presentation

### 2.1 Initial presentation and diagnosis

**Figure 1:**
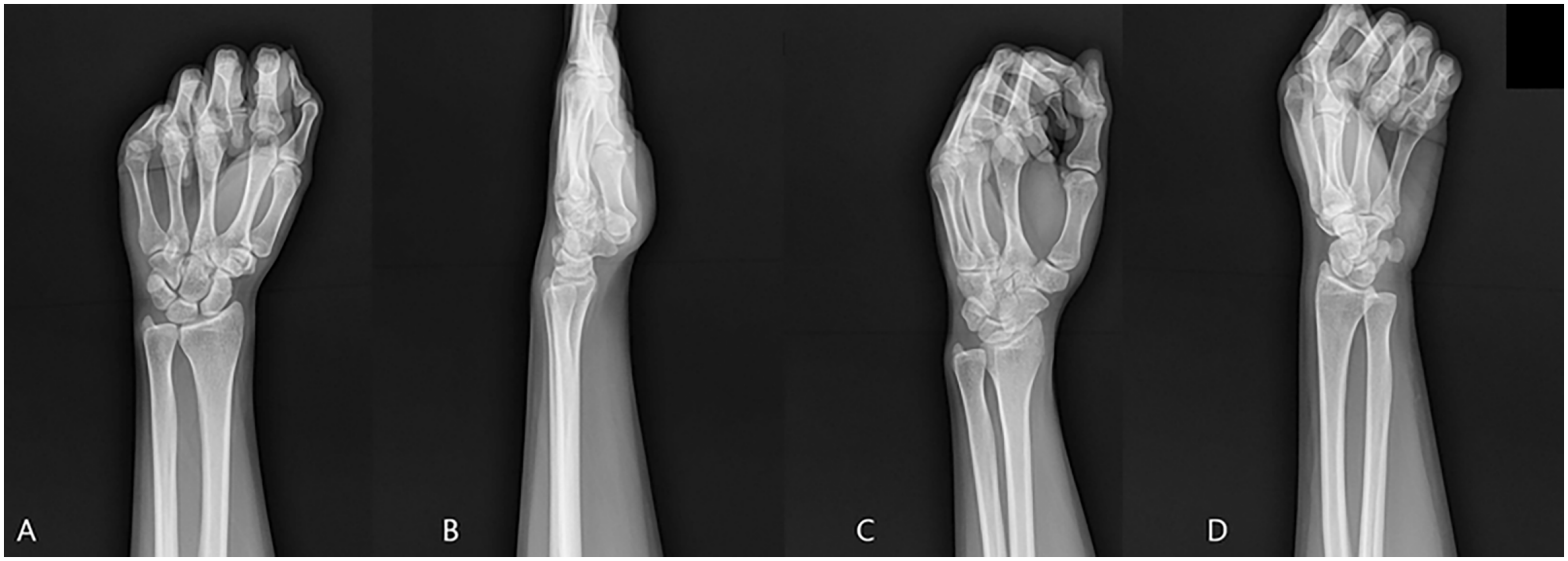
Initial radiographic series of the left wrist **(A)** Anteroposterior, **(B)** Lateral, **(C)** Internal Oblique, **(D)** External Oblique. No definitive fracture line is observed.

On June 29, 2019, a 29-year-old right-handed woman presented to our outpatient orthopedic clinic with acute-onset left wrist pain. The injury occurred when she fell onto her outstretched left hand after being pulled by another person. On initial physical examination, there was diffuse swelling, ecchymosis, and significant tenderness to palpation across the entire wrist, with a painfully limited range of motion in all planes. A standard four-view radiographic series of the left wrist was obtained, which did not reveal a definitive fracture line, dislocation, or other acute bony abnormality (Figure 1).

Based on predominant ulnar-sided tenderness on initial presentation, the patient was managed with a short arm ulnar gutter splint. After several days, the initial ulnar-sided pain and swelling resolved completely. However, this clinical improvement unmasked a persistent and now more prominent, exquisite tenderness localized to the radial side. Specifically, palpation of the anatomical snuffbox and the scaphoid tubercle elicited a sharp, focal pain (rated 8/10 on the Visual Analog Scale). Axial compression of the thumb also reproduced this pain, further heightening the clinical suspicion for an occult scaphoid fracture.

**Figure 2:**
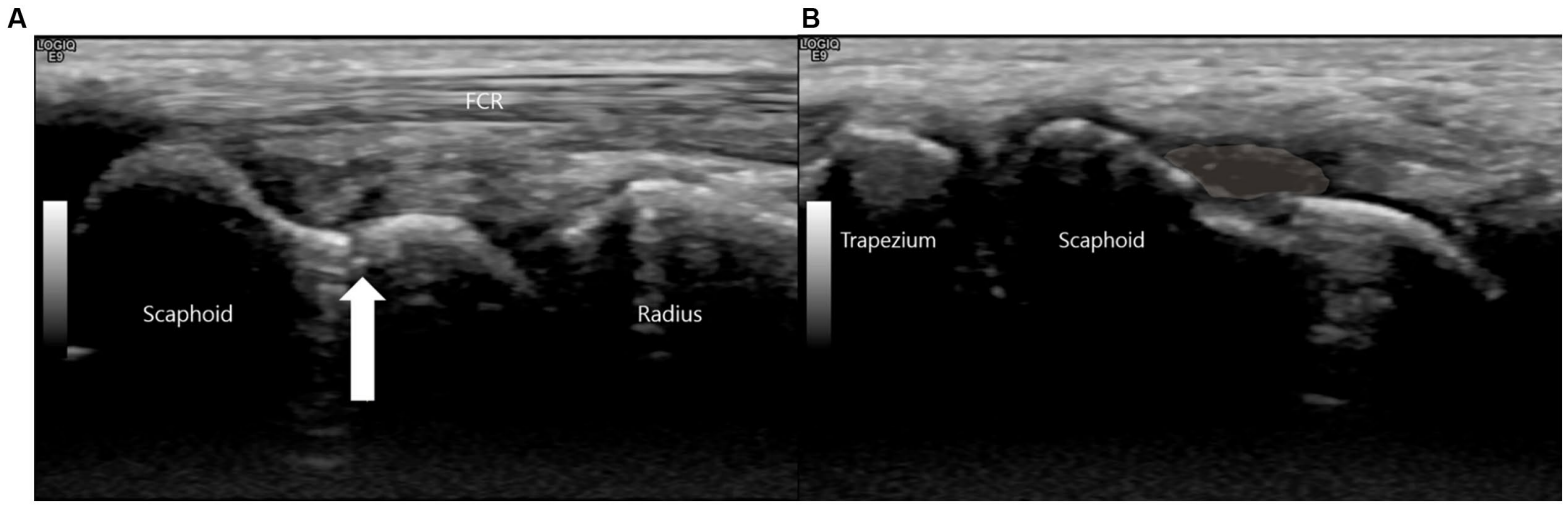
This is a high-resolution ultrasound image of the scaphoid waist. **(A)** The white arrow indicates the fracture site. **(B)** A hypoechoic collection consistent with a hematoma (shaded area) is noted superficial to the cortical breach.

Due to these persistent and highly localized signs, a high-resolution musculoskeletal ultrasound examination was performed at the point of care using a high-frequency linear transducer (18-5 MHz, LOGIQ E9, GE Healthcare). The point-of-care ultrasound examination began with a static assessment. With the wrist in slight ulnar deviation to maximize the longitudinal view of the scaphoid, a clear hypoechoic cortical breach was identified at the scaphoid waist. A small, overlying hypoechoic collection consistent with a hematoma was also noted (Figure 2).

This sonographic finding correlated precisely with the point of maximal tenderness elicited during the physical examination, confirming it as the anatomical source of the patient’s symptoms.

**Figure 3:**
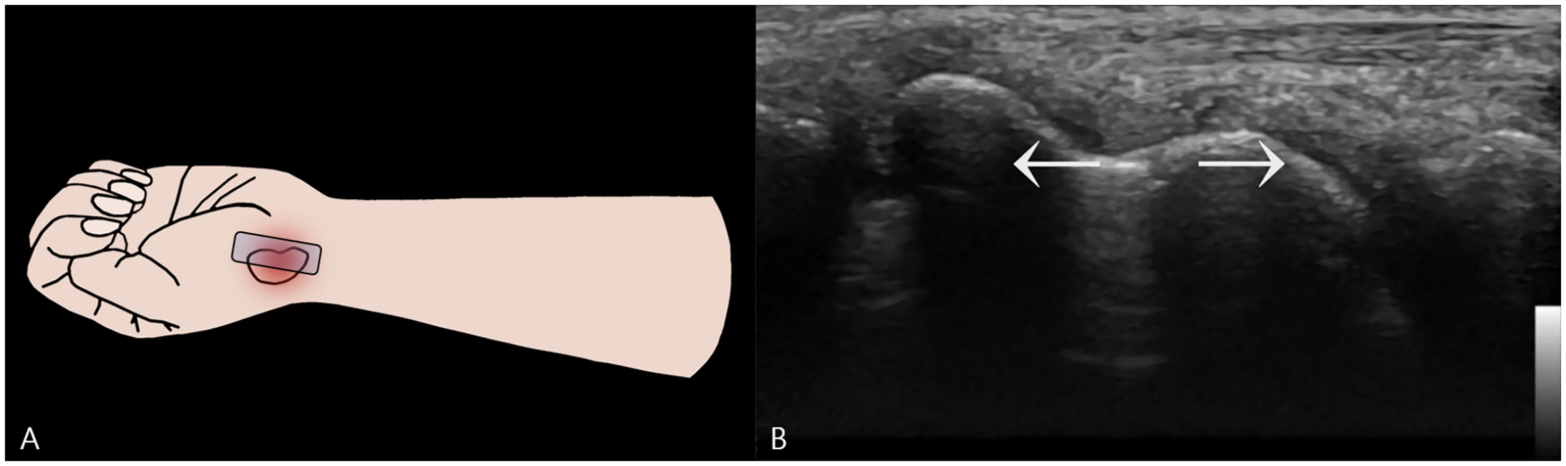
Dynamic Stress Examination of the Scaphoid Waist Fracture. **(A)** Schematic illustration showing the placement of the ultrasound probe longitudinally over the scaphoid waist. Focal pressure is applied directly onto the distal fragment using a “heel-toe” maneuver to assess for instability. **(B)** A two-dimensional ultrasound image captured during the application of dynamic stress. This image provides definitive evidence of mechanical instability by demonstrating a visible widening of the hypoechoic fracture gap.

To assess functional stability, a dynamic examination was performed. A dynamic stress maneuver is defined as a gentle, controlled “heel-toe” probe rocking that applies focal pressure across the fracture while maintaining a longitudinal view, used to elicit real-time widening of the fracture gap as evidence of mechanical instability (Figure 3). With the wrist in this position, the examiner applied this controlled maneuver. A definitive fracture line, visualized as a hypoechoic cortical breach, was identified. The purpose of this maneuver was to apply shear stress across the fracture site to challenge its integrity. In real-time, this stress resulted in a visible widening of the hypoechoic fracture gap, providing unequivocal evidence of micromotion and confirming the diagnosis of a mechanically unstable fracture (Supplementary Video S1).

Based on this definitive finding of instability, the management was immediately escalated. The initial splint was replaced with a rigid thumb spica short arm splint. This change was critical, as immobilizing the thumb is necessary to neutralize the forces acting on the scaphoid during thumb motion, thereby providing the robust stabilization required for an unstable waist fracture.

### 2.2 Follow-up and long-term outcome

Long-term follow-up was complicated by patient-specific circumstances that precluded surgical intervention, the standard recommendation for many unstable scaphoid fractures. Consequently, the patient was managed with prolonged conservative therapy. Radiographs obtained more than one-year post-injury remained inconclusive, showing no substantial interval changes and failing to provide clear evidence of healing.

**Figure 4:**
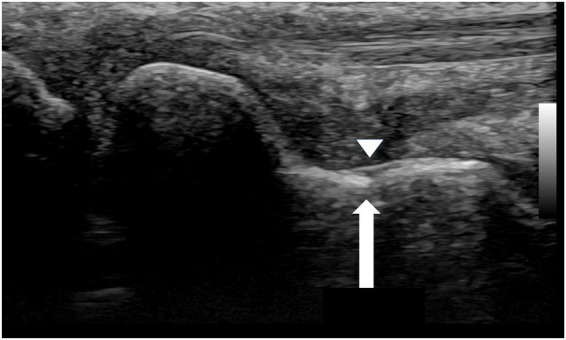
Follow-up ultrasound image of the waist region of the scaphoid (September 1, 2020). The white arrow indicates restoration of cortical continuity and formation of a bony bridge over the fracture site. The arrowhead demonstrates the bony callus formation.

This created a diagnostic dilemma, as the radiographic findings alone could have been misinterpreted as a persistent non-union. In contrast, the follow-up ultrasound examination provided a definitive assessment. It revealed early callus formation and restoration of cortical continuity across the scaphoid waist. Most critically, a repeat dynamic stress examination was performed, which revealed no widening of the former fracture site under stress, confirming that mechanical stability had been achieved (Figure 4).

This starkly contrasted with the ambiguous radiographs, demonstrating that the fracture had healed into a stable fibrous union with sufficient integrity to prevent micromotion, thereby allowing for a confident conclusion to the treatment plan.

## 3 Discussion

### 3.1 Diagnostic and functional insights from dynamic ultrasound

The accurate and timely diagnosis of scaphoid fractures is paramount to preventing long-term complications such as non-union and SNAC wrist (26). While static high-resolution ultrasound has been extensively validated for detecting cortical breaches (sensitivities and specificities exceeding 90%) (21–23, 27), the unique contribution of this case lies in demonstrating real-time, functional assessment of mechanical instability—a capability under-reported in the literature. We illustrate how this dynamic finding immediately altered the management algorithm in clinical practice, demonstrating value beyond anatomic characterization.

The dynamic maneuver described—applying focal pressure onto the distal fragment with “heel-toe” rocking motion—is a biomechanical stress test performed under direct visualization. Real-time gapping at the fracture site is a direct sonographic correlate of mechanical instability. This finding moves diagnosis from a purely anatomical question (“Is there a fracture?”) to a functional and prognostic one (“Is the fracture stable?”)—the single most important factor guiding treatment. Stable, non-displaced fractures can often be managed successfully with cast immobilization alone (union rates exceeding 90%), whereas unstable fractures have markedly higher non-union risk (approaching 50% or more when treated non-operatively) (28–30). The diagnostic accuracy of our approach was strengthened by multiple concordant findings: precise topographic concordance between maximal clinical tenderness and sonographic lesion, composite imaging signs (cortical breach and hematoma), dynamic gap widening confirming mechanical instability, direct management escalation, and follow-up dynamic testing showing loss of gapping confirming restored stability. This multimodal approach effectively ruled out false positives and anchored diagnosis in both anatomic and functional evidence. The ability to immediately risk-stratify these injuries at the point of care identified this patient for escalated management, with the sonographic diagnosis of instability justifying immediate application of rigid thumb spica splint rather than simple ulnar gutter splint.

Dynamic ultrasound provides real-time, functional information at the point of care, fundamentally distinguishing it from static imaging (CT, MRI, static US) (Table 1). While CT and MRI offer superior detailed anatomical assessment (8, 16), they are static snapshots from which instability must be inferred indirectly. Dynamic ultrasound directly demonstrates instability, transforming diagnosis into a functional biomechanical evaluation that directly answers the clinician’s most pressing question. This capability aligns with value-based care principles: rapid, low-cost, repeatable testing yielding high-impact clinical information. It prevents the common cycle of negative radiographs, uncertain immobilization, patient anxiety, and delayed diagnosis. Long-term follow-up in this case revealed another critical advantage: radiographs obtained over 1 year post-injury remained inconclusive, potentially suggesting non-union warranting surgery, while follow-up ultrasound revealed partial cortical restoration, callus formation, and, most importantly, absence of gapping on dynamic stress testing, confirming stable fibrous union. This highlights dynamic ultrasound’s unique ability to differentiate between symptomatic unstable non-union requiring surgery and asymptomatic stable fibrous union manageable conservatively (31). For fractures initially diagnosed by ultrasound, sonographic stability appears a more clinically relevant healing endpoint than complete radiographic consolidation.

**Table 1:** Diagnostic approaches for suspected scaphoid fracture with negative initial radiographs—strengths and typical use.

Modality	What it shows best	Typical strengths	Typical limitations	Role in workflow
Radiographs (4-view)	Gross fracture lines, displacement	Widely available, low cost	Early false-negatives common	First-line screen
CT	Cortical detail, displacement, morphology	Precise anatomic quantification; surgical planning	Radiation; cost	Structural clarification
MRI	Marrow edema, occult fractures	Highest sensitivity in early phase	Access; cost	Confirmatory when high suspicion persists
Static US	Cortical breach; superficial hematoma	Point-of-care; no radiation; high resolution	Operator-dependent; primarily structural	Rapid confirmation in clinic/ED
Dynamic US (this report)	Real-time mechanical instability (gap widening)	Functional assessment that directly informs management	Standardization & thresholds needed; operator-dependent	Decision pivot in POCUS-first pathway

Successful application requires several considerations. The technique is highly operator-dependent, requiring proficiency in wrist sonography and thorough understanding of scaphoid anatomy. High-frequency linear transducers (≥12 MHz) are essential for adequate spatial resolution (19, 20). The “heel-toe” pressure method, designed to create shear stress across the scaphoid waist, must be controlled to be diagnostic without causing undue pain or iatrogenic displacement. Standardization of dynamic ultrasound protocols represents a critical future development, with research focusing on defining optimal stress maneuvers for different fracture locations and establishing quantitative criteria for instability (e.g., millimetric thresholds for gap widening). This case report has notable strengths, including clear step-by-step illustration of dynamic technique and direct demonstration of its impact on patient management, with long-term follow-up providing insight into natural history of unstable fractures managed conservatively. However, conclusions are tempered by inherent single-case limitations and cannot be generalized. The technique’s operator-dependence and potential for iatrogenic injury with excessive stress require cautious, controlled technique application. Future prospective, multicenter studies are urgently needed to directly compare diagnostic accuracy of dynamic ultrasound against CT and MRI for assessing fracture stability (4, 15), establish inter-rater reliability among operators of varying experience, quantify millimetric thresholds for instability, and conduct formal cost-effectiveness analyses. Based on current evidence, we propose a modified diagnostic algorithm for suspected scaphoid fractures with negative initial radiographs: (1) Detailed clinical examination including anatomical snuffbox palpation and axial thumb compression; (2) If high clinical suspicion, proceed directly to point-of-care ultrasound—first static assessment to confirm cortical discontinuity; (3) If static ultrasound negative, scaphoid fracture is highly unlikely and immobilization can be avoided; (4) If static ultrasound positive, immediately perform dynamic stress testing to assess stability; (5) If dynamic ultrasound reveals instability, urgent orthopedic consultation and consideration of early surgical fixation is warranted; (6) CT or MRI is reserved for anatomic detailing in surgical planning or when ultrasound findings are equivocal. Implementation of this streamlined Clinical Exam → POCUS (Static + Dynamic) pathway could significantly reduce costs and patient morbidity associated with traditional “wait-and-see” approaches. For patients with wrist sprains but no fracture, negative ultrasound could immediately and confidently rule out scaphoid injury, preventing weeks of unnecessary cast immobilization and patient anxiety. For patients with confirmed unstable fractures, it ensures immediate, appropriate treatment, mitigating devastating long-term complications such as AVN and SNAC wrist.

## 4 Conclusion

This case report describes a patient with a radiographically occult scaphoid fracture in whom dynamic musculoskeletal ultrasound was instrumental for both initial diagnosis and the crucial assessment of mechanical stability. The ability to confirm instability in real-time at the point of care allowed for an immediate and appropriate escalation of conservative management. This case adds to the growing body of literature supporting the use of ultrasound in the evaluation of acute wrist trauma and specifically highlights the unique, value-added information provided by a dynamic examination. We believe that for experienced operators, dynamic MSK-US should be considered a primary imaging tool in the algorithm for suspected scaphoid fractures, offering a rapid, accurate, and radiation-free pathway to definitive diagnosis and optimized patient care.

## Data Availability

The original contributions presented in the study are included in the article/Supplementary material, further inquiries can be directed to the corresponding author.

## Supplementary material

Supplementary Video S1 Dynamic ultrasound examination of the scaphoid waist fracture.
